# Angiography-derived index of microvascular resistance in takotsubo syndrome

**DOI:** 10.1007/s10554-022-02698-6

**Published:** 2022-11-07

**Authors:** Gianluca Castaldi, Simone Fezzi, Maddalena Widmann, Micaela Lia, Francesca Rizzetto, Concetta Mammone, Sara Pazzi, Solange Piccolo, Verdiana Galli, Michele Pighi, Gabriele Pesarini, Daniele Prati, Valeria Ferrero, Roberto Scarsini, Domenico Tavella, Flavio Ribichini

**Affiliations:** Division of Cardiology, Department of Medicine, University of Verona, Piazzale Aristide Stefani 1, 37126 Verona, Italy

**Keywords:** Takotsubo syndrome, Quantitative flow ratio, Index of microvascular resistance, Coronary physiology

## Abstract

Coronary microvascular dysfunction (CMD) has been proposed as a key driver in the etiopathogenesis of Takotsubo syndrome (TTS), likely related to an “adrenergic storm” upon a susceptible microvascular circulation. The aim of our manuscript was to assess CMD in patients with TTS through the computation of the angiography-derived index of microcirculatory resistance (IMR) and its correlation with clinical presentation**.** Coronary angiograms of 41 consecutive TTS patients were retrospectively analyzed to derive angiography-based indices of CMD. Three indices (NH-IMRangio, AngioIMR and A-IMR) were calculated based on quantitative flow ratio. CMD was defined as an IMRangio value ≥ 25 units. The correlation between CMD and clinical presentation was then assessed. Median age was 76 years, 85.7% were women and mean left ventricular ejection fraction (LVEF) at first echocardiogram was 41.2%. Angiography-derived IMR was higher in left anterior descending artery (LAD) than circumflex and right coronary artery with either NH-IMRangio (53.9 ± 19.8 vs 35.8 ± 15.4 vs 40.8 ± 18.5, p-value < 0.001), AngioIMR (47.2 ± 17.3 vs 31.8 ± 12.2 vs 37.3 ± 13.7, p-value < 0.001) or A-IMR (52.7 ± 19 vs 36.1 ± 14.1 vs 41.8 ± 16.1, p-value < 0.001). All patients presented CMD with angiography-derived IMR ≥ 25 in at least one territory with each formula. Angiography-derived IMR in LAD territory was significantly higher in patients presenting with LVEF impairment (≤ 40%) than in those with preserved ventricular global function (NH-IMRangio: 59.3 ± 18.1 vs 46.3 ± 16.0 p-value = 0.030; AngioIMR: 52.9 ± 17.8 vs 41.4 ± 14.2, p-value = 0.037; A-IMR: 59.2 ± 18.6 vs 46.3 ± 17.0, p-value = 0.035). CMD assessed with angiography-derived IMR is a common finding in TTS and it is inversely correlated with LV function. The available formulas have a substantial superimposable diagnostic performance in assessing coronary microvascular function.

**Supplementary Information:**

The online version contains supplementary material available at 10.1007/s10554-022-02698-6.

## Introduction

Stress cardiomyopathy, known as Takotsubo syndrome (TTS), is a clinical condition that mimics acute myocardial infarction in terms of symptoms (chest pain) and electrocardiographic presentation (ST-T segment modifications, deep T-wave inversion) although in absence of significative epicardial coronary disease. The typical echocardiographic kinetic pattern (ipo-akinesia of apical segments with hyperkinesia of basal segments) involves a significant portion of myocardium but is usually associated with a modest release of myocardial injury markers [1–4].Despite its physio-pathological momentum is not well understood yet, the occurrence of a serum catecholaminergic storm induced by exaggerated sympathetic stimulation (emotional or physical trigger) acting on an abnormal and dysfunctional microvascular substrate is most likely involved [5].

The thermodilution-derived index of microvascular resistance (IMR) is the most commonly used surrogate to invasively assess coronary microvascular dysfunction (CMD) with established diagnostic and prognostic relevance in acute (ACS) and chronic coronary syndromes (CCS) [6–8] [9]. It is based on the sequential selective coronary injection of 3 ml cold saline boluses, generally in the left anterior descending artery (LAD), during both resting condition and after steady hyperaemia induction: mean transit time at rest and during hyperaemia are estimated and surrogate indices of coronary flow and microvascular resistance, such as IMR, can be derived*.* Invasive assessment of CMD in TTS has been previously reported in case reports and small case series [10–12]. However, its adoption in the clinical practice is globally low due to its costs, to the need for a dedicated pressure-wire and steady state hyperaemia. Moreover, the clinical relevance in the context of TTS has not been proven yet.

To overcome these limitations, the concept of IMR based on angiography has been developed. Angiography-derived IMR is based on the application of computational flow dynamics to the three-dimensional quantitative coronary angiography (QCA) [13] and it has been validated against invasive IMR and cardiac magnetic resonance in both ACS and CCS settings [14]. Several formulas for the computation of angiography-derived IMR have been then developed. However, a head-to-head comparison of these novel indices is not yet available.

In this study we sought to investigate the association of TTS with CMD through the computation of angiography-derived IMR, according to three validated formulas. Secondly, we aimed to assess the correlation of CMD with the clinical presentation. Finally, for the first time we provided a paired comparison of the three major formulas for the computation of IMR derived from angiography.

## Methods and materials

### Study population

Sixty-five consecutive patients admitted for TTS from January 2018 to August 2021 at the University Hospital of Verona were initially screened for inclusion in this retrospective observational study; between these, 24 patients were excluded because of technically not adequate coronary angiography (CAG) for QFR analysis. TTS diagnosis was defined according to the Revised Mayo Clinic [16] and InterTAK diagnostic criteria, as [1] a transient wall motion abnormality in the left ventricle beyond a single epicardial coronary artery distribution; [2] the absence of obstructive coronary artery disease, which could explain the wall motion abnormality; [3] new electrocardiographic abnormalities or elevation in cardiac troponin values; and [4] the absence of pheochromocytoma or myocarditis. CAG was undertaken for each patient during acute phase, according with international recommendations based on clinical presentation [15, 16]. Ultimately, QFR analysis was performed in 109 coronary vessels (41 patients), that were included in the final analysis. A detailed study flow-chart is provided in Supplementary Fig. 1.

The study was conducted in accordance with the ethical principles of the Declaration of Helsinki and all the patients included entered in the prospective clinical registry and provided their written consent for the anonymous collection of the data.

### Demographic and clinical variables

For each patient, demographic variables and cardiovascular risk factors were collected at admission. Electrocardiographic (EKG) abnormalities (i.e. ST elevation, ST depression, T-wave inversion, atrial fibrillation) were collected from the first EKG available. Peak levels of high-sensitivity troponin (hs-cTnT or hs-cTnI) were measured. To compare values of the two different indices of myocardial injury, the value of peak level divided by respective 99th percentile upper reference limit (URL) of the assay (hs-cTNT URL = 14 ng/L, hs-cTNI URL = 45 ng/L) was considered (cTnxURL) according to the international recommendation [16]. Creatinine value was collected at admission and creatinine clearance (CrCl) was calculated by Cockcroft-Gault formula. The left ventricular ejection fraction (LVEF) was assessed by echocardiography using the biplane Simpson method at admission. Percentage of inotropic and/or mechanical circulatory support was collected. All the patients underwent a follow-up transthoracic echocardiography, within one month from discharge.

### QFR analysis and angiography-derived IMR computation

The 3D-QCA analysis and QFR computation were performed by two independent, experienced, trained and certified different investigators (GC, SF) blinded to clinical presentation. A validated software (QAngio XA 3D version 3.2.48.8, Medis Medical Imaging Systems, Leiden, The Netherlands) was used. QFR computation was performed in agreement with the step-by-step procedure validated in previous studies. As per QFR protocol, two angiographic projections at least 25°apart were selected, avoiding foreshortening and overlap between the main vessel and side branches. In each projection, the end-diastolic frame was selected and one anatomical landmark was identified in both as the reference point. Subsequently, the proximal and distal points were selected, while vessel contours were automatically detected and manually corrected if necessary. The software reconstructed a 3D anatomical vessel model without its side branches for the 3D-QCA analysis and QFR computation. Vessel length was automatically calculated by the software, while the number of frames (Nframes) required for contrast dye to travel from the proximal to the distal reference was manually assessed. The frame-acquisition rate was set at 15 frames/second. From these values flow velocity and estimated hyperemic flow velocity (*Vhyp*) were derived [17]. All measures were collected at rest without necessity of hyperaemia induction. Therefore, angiography-derived IMR was calculated using the validated formulas [13, 14, 18–20], where Pa is the mean aortic pressure, cQFR the contrast quantitative flow-ratio and Vhyp the hyperemic coronary flow velocity:

1 $$1.\,NH - IMRangio = Pa \times cQFR \times \frac{Nframes}{{fps}}$$

2 $$2.\,AngioIMR = Pa - \left( {0.1 \times Pa} \right)] \times cQFR \times (Vessel lenght/Vhyp)$$

3 $$3.\,A - IMR = \frac{{Pa *\left( {vessel lenght/flow velocity} \right) * \left[ {\left( {1.35*cQFR} \right) - 0.32} \right]}}{100}$$

**Fig. 1:**
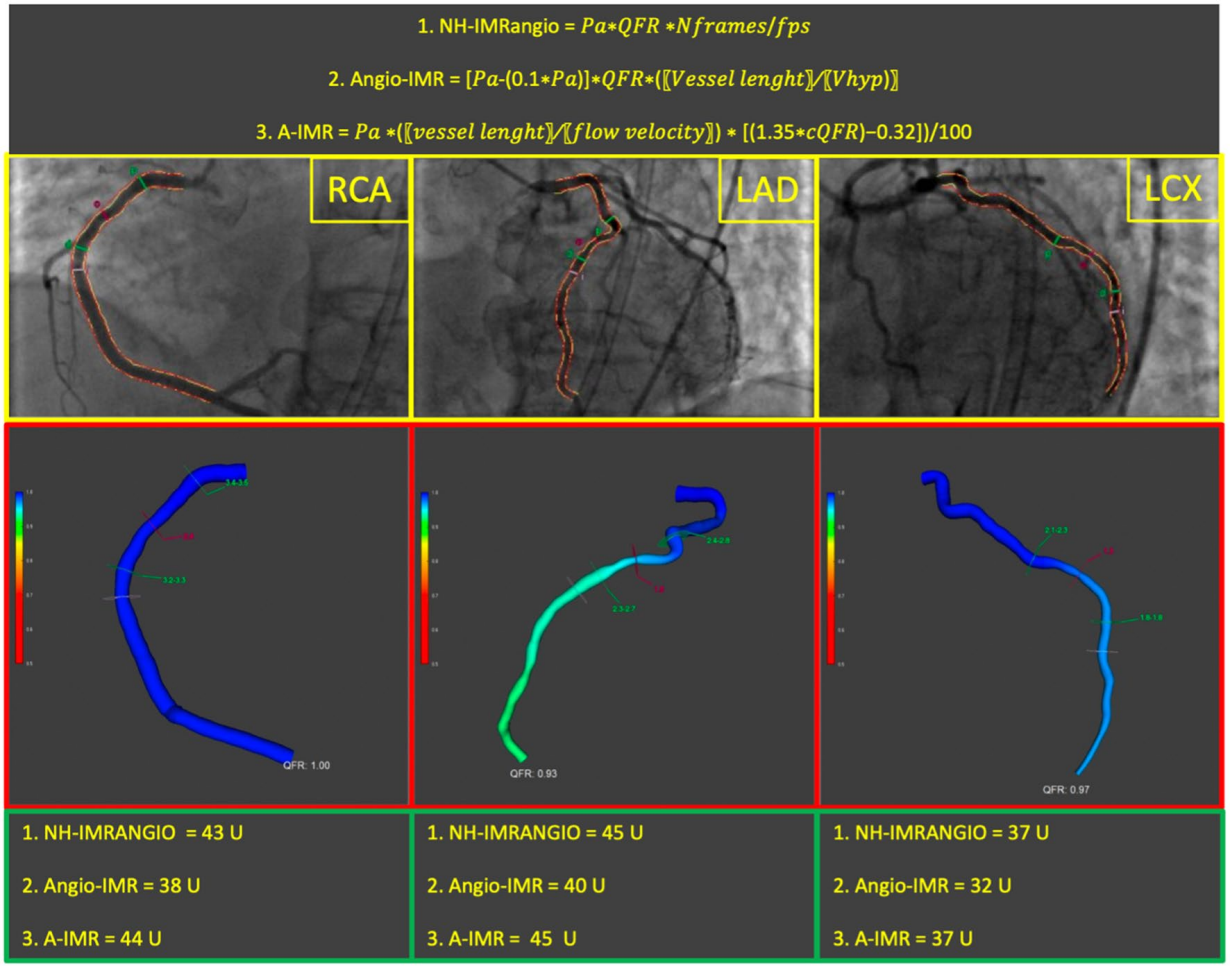
Step-by-step angiography-derived IMR computation from QFR. Firstly, 2D-QCA analysis of target vessel is performed; secondly, 3D-QCA model simulation is automatically derived and vessel QFR is computed by the software. Finally, IMR according to the respective formulas is calculated, *cQFR* contrast-QFR; *fps* frame per second; *IMR* index of microvascular resistance; *LAD* left anterior descending; *LCX* left circumflex; *N* number; *Pa* arterial pressure; *QFR* quantitative flow ratio; *RCA* right coronary artery

A comprehensive illustration of the computation of angiography-derived IMR through the three different formulas is provided in Fig. 1.

### Objective of the study

The objectives of our study were: (1) to investigate the incidence of coronary microvascular disfunction in TTS through angiography-derived IMR computation; (2) to assess the correlation of angiography-derived IMR with clinical variables, such as LVEF and laboratory biomarkers; (3) to compare the diagnostic performance of the three different IMR computation formulas in this clinical setting.

### Statistical analysis

Clinical and demographic variables are presented as means with standard deviation for continuous variables with normal distribution and as medians with interquartile range for continuous variables with non-normal distribution. The normality of the distribution was assessed by Shapiro–Wilk test and QQ plots analysis. Categorical variables are presented as counts and respective percentages. For continuous variables, they were analysed by t-test or ANOVA in the case of a normal distribution and by Mann–Whitney U-test test in the case of a non-normal distribution. Categorical variables were analysed by Fisher's exact test or χ^2^ test. NH-IMRangio according to each formula for LAD, left circumflex (LCX) and right coronary (RCA) artery was computed. Angiography-derived IMR ≥ 25 was considered as cut-off for coronary microvascular dysfunction definition. To study the correlation of CMD with clinical and laboratory variables, the highest angiography-derived IMR value between the three vessels was chosen. Correlations were analysed using Pearson test if they presented a normal distribution and Spearman test if they had a non-normal distribution.

Final agreement evaluation between the three formulas was conducted according to Bland–Altman plot analysis. Statistical analysis was performed using SPSS 26.0 (Inc Chicago, Illinois) and Prism 9.0 (GraphPad Software, San Diego, California). A p value < 0.05 was considered statistically significant.

## Results

### Demographic and clinical characteristics

The population analyzed was prevalently female (85.7%) with a median age of 76 years (67.5–81.5) and a mean body mass index of 23.1 ± 3.1 kg/m^2^. Most of the patients presented with medical history of arterial hypertension (23, 65.7%) while only three patients (8.6%) were diabetic and 13 (31.7%) had chronic kidney disease with a global mean CrCl of 64.26 ± 22.4 ml/min. Two patients had previous history of coronary artery disease (CAD) and 8 (18.5%) had documented peripheral artery disease. At admission, most of the patients presented with Killip class one (26, 74.3%) while three patients (8.1%) presented with cardiogenic shock (Killip class four); five patients (12.8%) required inotropic support and only in one patient mechanical circulatory support with intra-aortic balloon pump was necessary during the hospital stay. One patient (2.6%) had a stroke and died during hospitalization. 11 patients (25.7%) presented with ST-segment elevation, two patients (5.7%) with ST-segment depression and 13 patients (37.1%) with deep T-wave inversion. According to InterTAK diagnostic score, potential emotional or clinical triggers were investigated: in 24 patients (58.5%) a clear physical trigger (e.g. trauma, infection, severe pain, etc.) was identified, while in nine patients (22%) the trigger was emotional. Mean LVEF at the first echocardiogram was 41.2 ± 10% with 23 patients (56%) presenting with LV dysfunction defined as LVEF < 40%. CAG was performed within 24 h in 27 cases (66%) and within 72 h in 34 cases (83%).

Serial measurements of hs-cTn were collected with median cTnxURL of 123 (20–110). All the clinical variables are reported in Table 1.

**Table 1:** Baseline characteristics and clinical variables of the population investigated (n = 41) along with comparison after stratification by LVEF ≤ 40% (n = 23) or > 40% (n = 18)

Variable	All patients	LVEF ≤ 40%	LVEF > 40%	p-value
	(n = 41)	(n = 23)	(n = 18)	
Age (years)	76 (67.5–81.5)	75 (69–82)	77 (69–82)	0.533
Female sex	36 (85.7%)	21 (91.3%)	14 (82.4%)	0.716
Arterial hypertension	24 (58.5%)	14 (60.9%)	10 (58.8%)	0.896
Smoking history	7 (20%)	3 (13%)	4 (22.2%)	0.299
Body mass index (kg/m^2^)	23.1 ± 3.1	23.0 ± 2.8	23.5 ± 3.6	0.694
BSA (mosteller)	1.59 ± 0.29	1.6 ± 0.1	1.7 ± 0.1	0.246
Diabetes	3 (8.1%)	1 (4.3%)	2 (11.8%)	0.573
Dyslipidemia	20 (48.8%)	11 (47%)	9 (50%)	0.762
Coronary artery disease	2 (5.1%)	1 (4.3%)	1 (5.5%)	0.791
Peripheral artery disease	8 (18.5%)	2 (8.7%)	6 (33%)	**0.028**
Chronic lung disease	4 (9.8%)	3 (13%)	1 (5.5%)	0.593
Atrial fibrillation	4 (9.8%)	3 (13%)	1 (5.9%)	0.593
CKD (CrCl < 60 ml/min)	13 (31.7%)	8 (34.8%)	5 (27.8%)	0.901
CrCl (ml/min)	64.26 ± 22.4	64.3 ± 23.9	64.2 ± 20.9	0.991
Killip class				
Class1	26 (70.3%)	16 (69.5%)	10 (55.5%)	0.514
Class2	8 (21.6%)	5 (21.7%)	4 (22.2%)	0.999
Class 3	0	0	0	–
Class 4	3 (8.1%)	2 (8.6%)	1 (5.5%)	0.268
TnxURL	123 (20–110)	32 (15–96)	53 (32–113)	0.263
ST-elevation	11 (25.7%)	7 (30.4%)	4 (22.2%)	0.199
ST-depression	3 (8.1%)	1 (4.3%)	2 (11.1%)	0.268
T-wave inversion	13 (37.1%)	6 (26.1%)	7 (38.9%)	0.116
QTc max (msec)	514.9 ± 40.8	511.9 ± 40.8	523.3 ± 43	0.412
LVEF (%)	41.2 ± 10	–	–	–
Critical state				0.306
Use of inotropic agents	5 (12.8%)	4 (17.3%)	1 (5.5%)	
IABP	1 (2.6%)	0	1 (5.5%)	
NIV/IOT	3 (7.3%)	2 (8.7%)	1 (5.5%)	0.760
Time to CAG				0.137
CAG ≤ 24 h	27 (66%)	17 (73.9%)	10 (56%)	
CAG ≤ 72 h	34 (83%)	18 (78.2%)	16 (88.8%)	
Death	1 (2.6%)	1 (4.3%)	0	0.999
Trigger				
Physical trigger	24 (58.5%)	14 (60.9%)	10 (55.5%)	0.759
Emotional trigger	9 (22%)	5 (21.7%)	4 (22.2%)	0.999
No apparent trigger	8 (19.5%)	4 (17.4%)	4 (22.2%)	0.713
NH-IMRangio LAD	53.9 ± 19.8	59.3 ± 18.1	46.3 ± 16.0	**0.030**
Angio-IMR LAD	47.2 ± 17.3	52.9 ± 17.8	41.4 ± 14.2	**0.037**
A-IMR LAD	52.7 ± 19.0	59.2 ± 18.6	46.3 ± 17.0	**0.035**

*BSA* body surface area; *CKD* chronic kidney disease; *CrCl* creatinine clearance; *LVEF* left ventricular ejection fraction; *hsTnxURL* high-sensitivity troponin upper reference limit; *IABP* intra-aortic balloon pump; *NIV/IOT* non-invasive/invasive ventilation; *CAG* coronary angiography; *LAD* left anterior descending artery. Statistically significant p-values are evidenced in bold

### Angiography-derived IMR analysis

QFR analysis and angiography-derived IMR calculation was feasible for all the three main vessels in 31 patients (75.6%), while in 39 patients (95%) calculation was feasible for two vessels and in the reminder only LAD was evaluable. Vessel QFR was lower in LAD compared to LCX and RCA respectively (0.91 ± 0.07 vs 0.97 ± 0.03 vs 0.95 ± 0.04; p < 0.0001). Angiography-derived IMR assessed in the LAD was higher than in LCX and RCA with either NH-IMRangio (53.9 ± 19.8 vs 35.8 ± 15.4 vs 40.8 ± 18.5, p-value < 0.001), Angio-IMR (47.2 ± 17.3 vs 31.8 ± 12.2 vs 37.3 ± 13.7, p-value < 0.001) or A-IMR (52.7 ± 19 vs 36.1 ± 14.1 vs 41.8 ± 16.1, p-value < 0.001). CMD defined as an angiography-derived IMR ≥ 25 in at least one territory was present in all the patients. Angiography-derived IMR results are provided in Table 2.

**Table 2:** QFR analysis of the three main vessels: comparison between the three formulas showed non-significant difference between each other (p > 0.05)

Vessel	Vessel lenght (mm)	Vessel QFR	NH-IMRangio	AngioIMR	A-IMR	p-value
LAD	100 ± 23	0.91 ± 0.07	53.9 ± 19.8	47.2 ± 17.3	52.7 ± 19	0.338
LCX	69 ± 15	0.97 ± 0.03	35.8 ± 15.4	31.8 ± 12.2	36.1 ± 14.1	0.430
RCA	101 ± 24	0.95 ± 0.04	40.8 ± 18.5	37.3 ± 13.7	41.8 ± 16.1	0.531
MEAN	–	–	38.2 ± 13.4	34.3 ± 12.0	37.8 ± 14.2	0.288

*LAD* left anterior descending; *LCX* left circumflex; *RCA* right coronary artery

### Angiography-derived IMR and clinical presentation

Angiography-derived IMR in the LAD territory demonstrated a moderate inverse correlation with LVEF. This was confirmed using all the different formulas (NH-IMRangio: r = − 0,3485, Rho = 0,1214, p = 0,0256; Angio-IMR: r = − 0,3513; Rho = 0,1234, p = 0,0256; A-IMR: r = − 0,3326, Rho = 0,1106, p = 0,0336). No statistical significant association was observed between mean value of IMR assessed in the three vessels and LVEF (NH-IMRangio: r = − 0.005, p-value 0.974; Angio-IMR: r = − 0.096, p-value = 0.953; A-IMR: r = − 0.044, p-value = 0.789).

No significant correlation was observed between angiography-derived IMR LAD and hsTnxURL (NH-IMRangio: r = − 0.008, p-value = 0.963; Angio-IMR: r = − 0.143, p-value = 0.385; A-IMR: r = − 0.143, p-value = 0.385). Angiography-derived IMR in the LAD territory was significantly higher in patients with LVEF ≤ 40% than patients with preserved LVEF (NH-IMRangio 59.3 vs 46.3, p. value = 0.030; Angio-IMR 52.9 vs 41.4, p-value = 0.037; A-IMR 59.2 vs 46.3, p-value = 0.035).

**Fig. 2:**
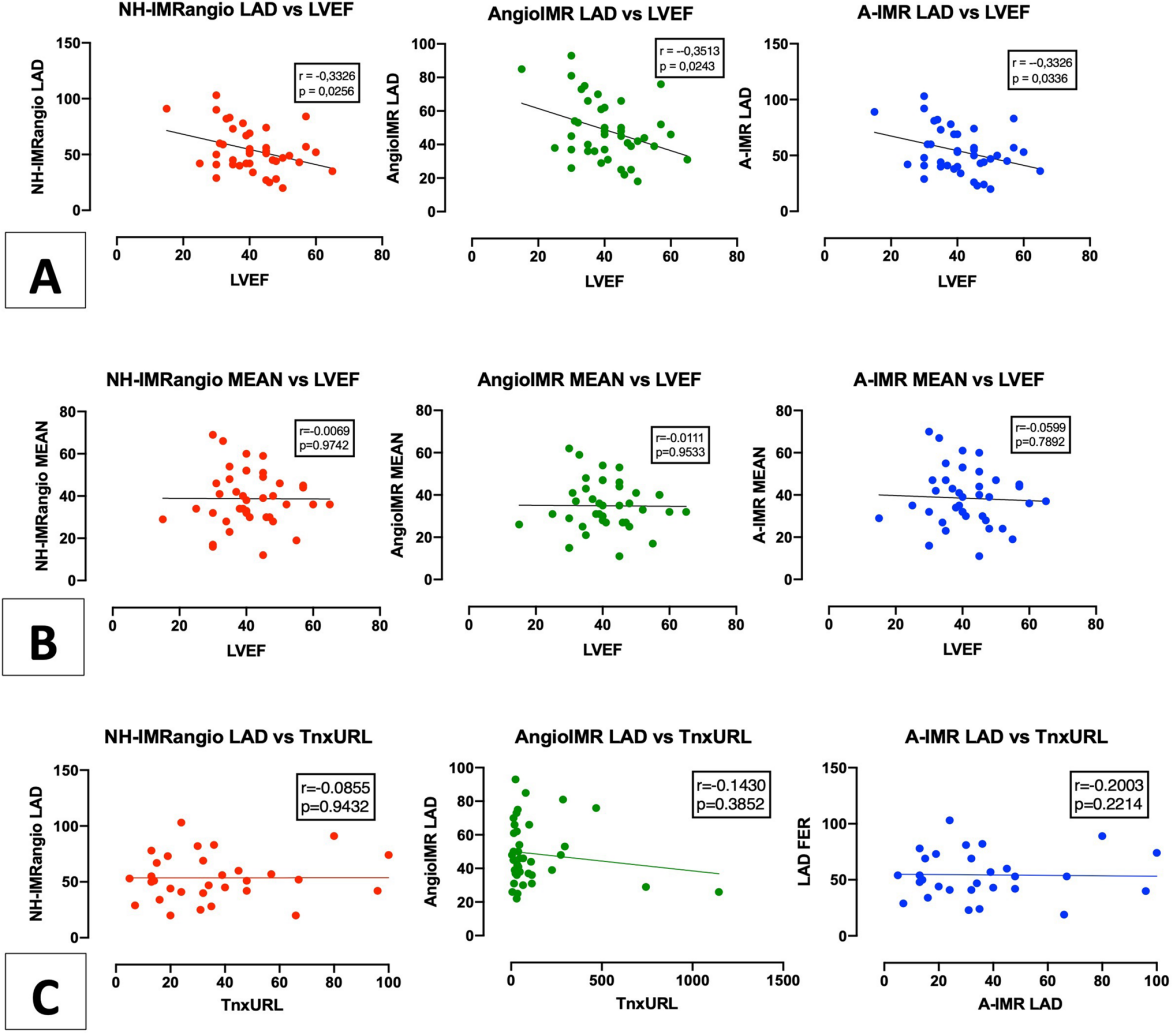
Correlation of angiography-derived IMR in the LAD territory with the three different formulas and LVEF (**A**) and TnxURL (**C**); correlation of global IMR MEAN according to three formulas and LVEF (**B**). *IMR* index of microvascular resistance; *LAD* left anterior descending; *LCX* left circumflex; *LVEF* left ventricular ejection fraction; *RCA* right coronary artery; *TnxURL* high-sensitivity troponin upper reference limit

A comprehensive illustration of correlations between angiography-derived IMR, LVEF and hsTnxURL is provided in Fig. 2.

### Angiography-derived IMR formulas comparison

**Fig. 3:**
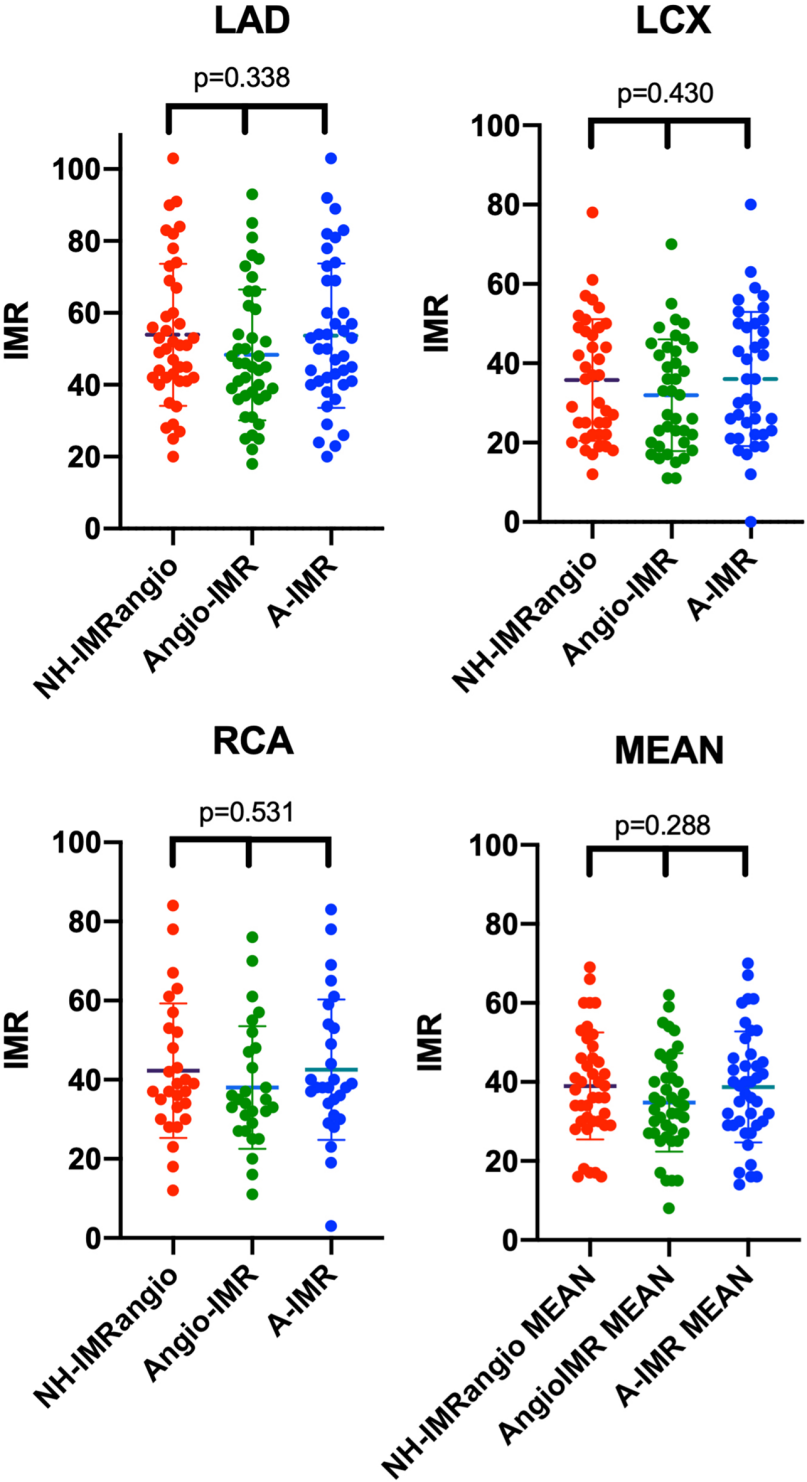
Scatter plot of angiography-derived IMR for each main epicardial vessel (LAD, LCX, RCA) and mean global value with each formula (NH-IMRangio, AngioIMR, A-IMR). IMR: index of microvascular resistance; *LAD* left anterior descending; *LCX* left circumflex; *RCA* right coronary artery

**Fig. 4:**
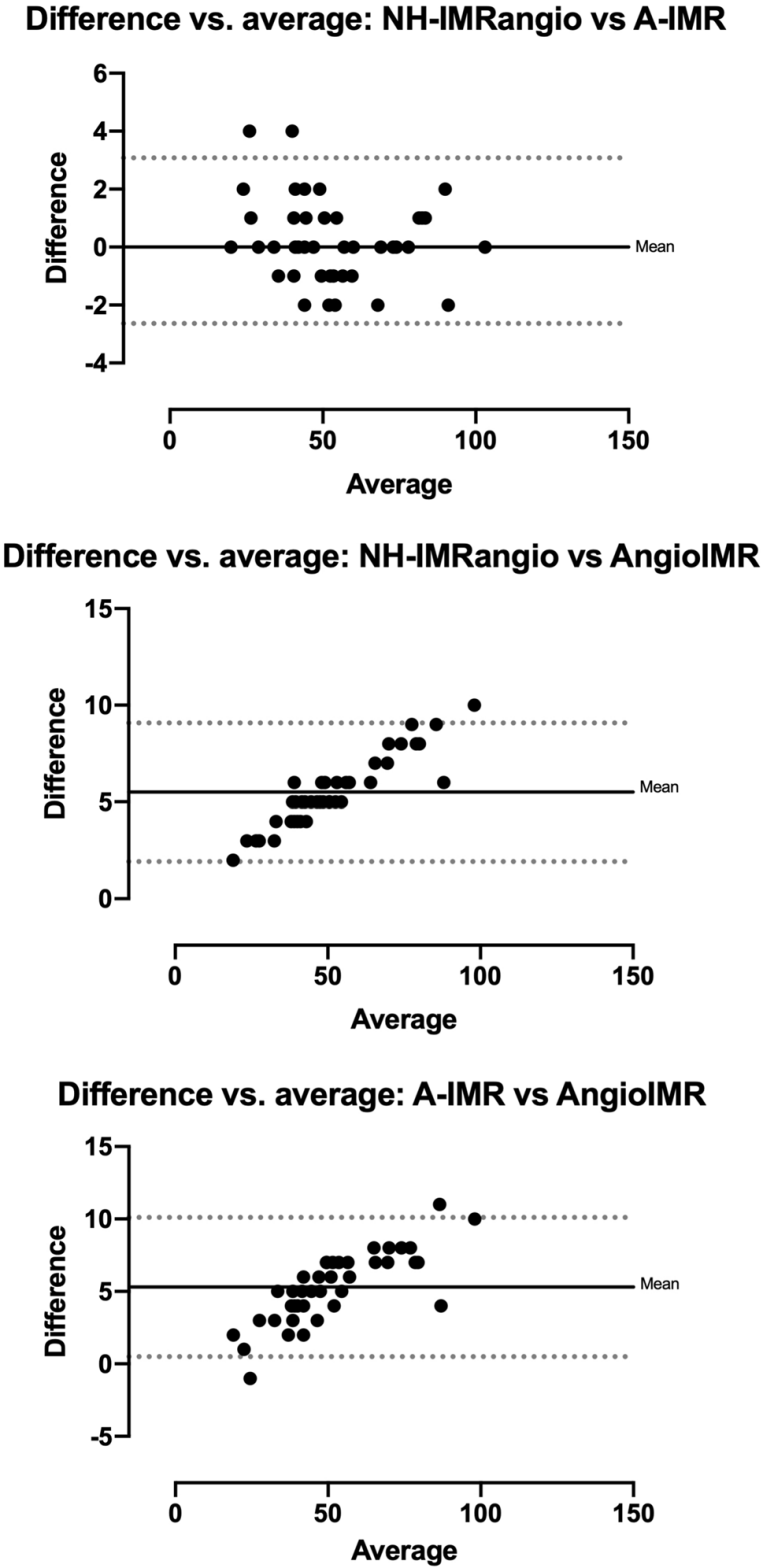
Bland–Altman plot analysis (difference vs average) of the agreement between the three formulas. *IMR* index of microvascular resistance

There was no statistical difference in assessment of microvascular function between the three formulas for each vessel (Fig. 3). Bland–Altman analysis showed substantial agreement between NH-IMRangio and A-IMR, while Angio-IMR showed consistently lower values (~ 5 units) against the other two formulas, across the whole range of IMR values, as provided in Fig. 4.

## Discussion

The main findings of our analysis are the following:

- 1 Coronary microvascular resistance assessed using angiography-derived IMR was significantly augmented in patients presenting with TTS;
- 2 Angiography-derived IMR computed in the LAD territory was inversely correlated with left ventricular systolic function;
- 3 The three formulas showed superimposable performance in assessing angiography-derived IMR.

**Fig. 5:**
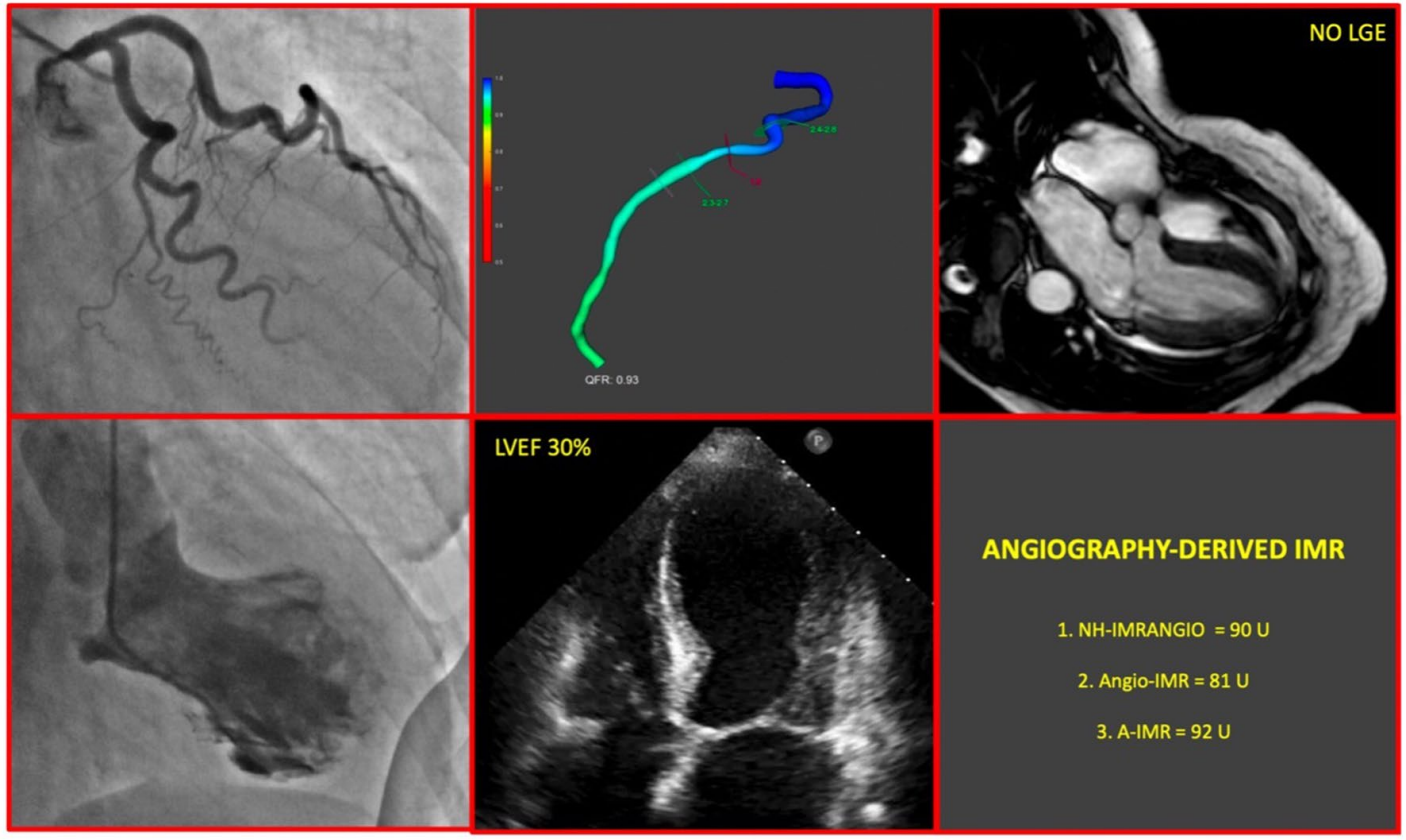
Multiparametric and multimodality approach to TTS. *CMD* coronary microvascular disfunction; *IMR* index of microvascular resistance; *LGE* late gadolinium enhancement; *LVEF* left ventricular ejection fraction

IMR does not represent a direct measurement of microvascular resistance, but is a derived surrogated index associated with clinical outcome in several clinical settings [14, 21–25]. Despite binary cutoffs do not properly express the nature of coronary physiology as a continuum, they are useful for theragnostic purposes. Stable patients without coronary obstructive disease generally have IMR values < 25 units [26]. Consistently, these patients tend to show normal myocardial perfusion reserve index on cardiac magnetic resonance while patients without coronary obstructive disease but IMR ≥ 25 units showed impaired myocardial perfusion reserve index, similarly to ischemic myocardium of patients with significative coronary artery disease [27]. In accordance with these and more data produced in the literature, the last EAPCI consensus document on ischemia with no obstructive coronary arteries, defines values of IMR ≥ 25 units as indicative of CMD [9]. More data are available in the setting of MI, and especially in patients presenting with ST-elevation MI, elevated IMR values correlate to poor microvascular reperfusion, with limited salvage and worse recovery of infarct size: in this population IMR > 40 units derived after primary percutaneous coronary intervention correlate with adverse left ventricular remodeling, but also with hard clinical outcomes like death, heart failure readmission, and major adverse cardiac event [6, 28]. Example of a plausible multiparametric and multimodality approach toward a patient-tailored treatment in TTS is shown in Fig. 5.

In TTS the evaluation of microcirculation has reported inconclusive results and no specific IMR cut-off is available. The precise pathophysiology of TTS has not been completely elucidated yet although a number of different mechanisms have been proposed. According to the most accredited rationale, a hyper-activation of the sympathetic nervous system occurs, inducing a neurohormonal catecholaminergic storm. This impacts on pre-existing microvascular abnormalities, determining unbalance between inappropriate α-mediated vasoconstriction and inadequate β2-mediated vasodilation. External microvascular compression due to myocardial oedema and inflammation-related cell accumulation and to increased intracardiac pressures may also contribute to the microvascular perfusion abnormalities [5, 29]. In accordance with the most plausible pathophysiological momentum subtending TTS, a prevalent functional microvascular impairment is expected compared to ST-segment elevation MI where a profound structural microvascular deterioration has been proved also in the acute phases.

The invasive study of coronary microvasculature in patients with TTS using IMR has been generally limited to the LAD, due to technical and procedural issues (e.g., invasive vessel wiring, hyperaemia induction, prolonged procedural times). Nonetheless non-invasive angiography derived IMR calculation allows to easily and rapidly assess the microcirculation status of all the three main epicardial coronary arteries, exploring global microvascular function. In normal coronary arteries, according to the laws of fluid dynamics, resistance increases across the branching structure of the coronary tree, because coronary flow decreases while the driving pressure remains virtually unchanged. Theoretically, IMR values could be influenced by the amount of myocardial mass subtended by the analyzed vessel, similarly to what has been proposed for FFR: this concept have been tested by Mauro Echavarría–Pinto et al., in CAD patients with intermediate stenosis, showing that indeed the amount of myocardium subtending to a coronary stenosis is inversely associated with IMR values [30]. In TTS patients however, according to what our findings suggest, CMD occurs globally into all the territories of coronary distribution but probably not with an uniform intensity: while some mechanisms can potentially induce CMD in all the three coronary artery territories (e.g., myocardial oedema secondary to intramyocardial catecholamine inflammatory process) others (e.g., myocardial stunning with external compression of micro-circulation and/or focal neurogenic-induced epicardial or microvascular spasm) are likely more pronounced in LAD territory. Thus, a global left ventricular involvement might be theorized in this scenario despite higher values of angiography derived IMR were observed in the LAD territories.

Interestingly, in our manuscript we showed a moderate correlation between CMD and left ventricular function reduction: while the relevance of such finding in terms of clinical presentation, management and long-term prognosis must be further investigated, concordant results have been publishing by other colleagues. In a recent manuscript by Jordi Sans-Roselló et al., a similar non-invasive microvascular assessment by means of NH-IMRangio derivation was performed in a large cohort of TTS patients. Concordantly to our analysis, a cut-off value of 25 units was chosen for the definition of CMD and a good correlation with LVEF was underlined, together with other variables (e.g., NTproBNP) not captured by our study [31].

However, to our knowledge, this is the first manuscript evaluating and directly comparing the performance of three different validated formulas for non-invasive angiography-derived IMR computation. IMRangio and its non-hypaeremic counterpart (NH-IMRangio) were the first formulas presented in the field of angiography-derived microcirculatory assessment and those with the largest published literature so far [13]. Subsequently, A-IMR and IMRangio were proposed by other groups. In the A-IMR formula a correction for collateral circulation is introduced, based on the Yong’s formula to measure corrected IMR (IMRcorr) [32]. The Yong’s formula takes into account theoretical variation of Pd in presence of significant epicardial stenosis, obviating the need for coronary wedge pressure measurement [14, 19]. AngioIMR differentiates from the other two formulas for the implementation of a correction factor for the estimation of coronary blood pressure during steady-state hyperemia. This assumption was based on the evidence of an average decrease in blood pressure of 10 mmHg during steady state adenosine-induced hyperaemia [33]. Consistently, the Bland–Altman plot showed systematic lower values of AngioIMR compared with the other angiography-derived IMR values. Without the possibility to compare angiography-derived indices with true invasive IMR values, we can not predict which formula gets closer to the actual invasive values of microvascular resistance however, in our analysis, this mathematical difference was not linked to a true clinical difference.

### Limitations

First of all, the retrospective nature of the analysis and the relative small sample size might have affected the inferential power of the study. Secondly, despite angiography-derived IMR has been validated as a pressure-wire-free alternative to IMR for the evaluation of coronary microvasculature it is indeed only a surrogate of the direct invasive thermodilution assessment, and it has not been extensively validated in TTS patients.

Moreover, we applied an angiography derived IMR > 25 units as cut-off that has been shown to be reliable in stable conditions or in the setting of non ST-segment elevation ACS [14]. In the setting of STEMI the cut-off point for prognostically significant CMD has been defined as an IMR > 40. However in the latter, CMD is mainly structural, as microvascular obstruction component is prevalent (e.g.: microthrombi, leukocyte adhesion and Neutrophil Extracellular Traps formation) whereas in the setting of Takotsubo CMD is believed to be primarily functional, as it is linked to endothelial dysfunction associated to the catecholaminergic cascade. Thus, the widely spread cut-off point of 25 seems to be reasonable and has been already proposed in the few previous experiences within this peculiar scenario [31, 34]. Nonetheless, only non-hyperemic measurements were performed in this study and comparison with invasive pressure-wire based IMR was not available. For this reason, no specific cut off was identified to predict clinically significant CMD and further larger studies are warranted.

Notably, coronary tortuosity has been reported to be more frequent in TTS as compared to matched controls. This anatomical aspect has been shown to impact both on pressure-wire derived and on angiography-derived epicardial physiology, leading to an underestimation of coronary flow. Whether this condition might have affected also angiography-derived IMR computation can not be excluded, and deserves further analysis. Nonetheless, cases with severe tortuosity were excluded from the analysis.

Finally, no data on the long-term trajectory of microvascular function were available: although a progressive recovery together with the LVEF improvement might be expected and has been previously reported, no angiographic follow-up was scheduled as not considered clinically advantageous in this peculiar scenario.

## Conclusions

This study demonstrated the presence of global dysfunction of the coronary microvasculature in patients with TTS. High values of angiography-derived IMR measured in the LAD territory were associated with TTS presentation with left ventricular systolic dysfunction.

## Supplementary Information

Below is the link to the electronic supplementary material.

Supplementary file1 (DOCX 31 KB)

## Abbreviations

ACS: Acute coronary syndrome
CAD: Coronary artery disease
CAG: Coronary angiography
CCS: Chronic coronary syndrome
CMD: Coronary microvascular dysfunction
LCX: Circumflex artery
IMR: Index of microvascular resistance
LAD: Left anterior descending artery
LVEF: Left Ventricular ejection fraction
MI: Myocardial infarction
QCA: Quantitative coronary angiography
QFR: Quantitative flow ratio
RCA: Right coronary artery
TTS: Takotsubo syndrome
