# Supplementary material for: Angiography-derived index of microvascular resistance in takotsubo syndrome
Source: Int J Cardiovasc Imaging. 2022 Nov 7;39(1):233–44. doi: 10.1007/s10554-022-02698-6 (PMC9813145; doi:10.1007/s10554-022-02698-6)
Supplement: Supplementary file 1 — Supplementary file1 (DOCX 31 KB) [file 10554_2022_2698_MOESM1_ESM.docx]

**Supplementary Fig 1:** Flowchart with exclusion criteria for QFR analysis

From January 2018 to August 2021:

**65 pts** with TTS
(enrolled in the local prospective registry)

Final cohort analyzed **41 pts** (total of 123 coronaries available for analysis)

Final **109** coronaries were analyzed with QFR software:

- 41 LAD
- 39 LCX
- 29 RCA

**24 pts** excluded due to technical issues:

- No isocenter data
- No DICOM available
- Poor imaging quality

**14 coronaries** were not adequately acquired for analysis:

- Single projection acquired (12 cases)
- Severe tortuosity with excessive overlap (2 cases)

**Supplementary Table 1:** Multivariate linear regression models analysis

| **NH-IMRangio** | | | | |
| --- | --- | --- | --- | --- |
|  | Unstandardized coefficients | | Standardized coefficient | |
|  | Beta | CI | Beta | p-value |
| Intercept | 53.85 | 45.33-62.36 | -- | <0.001 |
| DM | -15.0 | -38.2-8.2 | -0,21 | 0.197 |
| CAD | 33.65 | 5.65-61.64 | 0.39 | **0.020** |
| CKD | 0.22 | -13.42-13.87 | 0.01 | 0.973 |
| R=0.46, R^2^=0.22, Adjusted R^2^=0.14, F= 2.83, p= 0.054 | | | | |
| **AngioIMR** | | | | |
|  | Unstandardized coefficients | | Standardized coefficients | |
|  | Beta | CI | Beta | p-value |
| Intercept | 47.88 | 39.76-56.01 | -- | <0.001 |
| DM | -12.73 | -34.86-9.41 | -0.19 | 0.250 |
| CAD | 32.61 | 5.90-59.32 | 0.40 | **0.018** |
| CKD | -0.24 | -13.26-12.79 | -0.01 | 0.971 |
| R=0.46, R^2^=0.21, Adjusted R^2^=0.14, F= 2.79, p= 0.057 | | | | |
| **A-IMR** | | | | |
|  | Unstandardized coefficients | | Standardized coefficients | |
|  | Beta | CI | Beta | p-value |
| Intercept | 53.20 | 44.37-62.03 | -- | <0.001 |
| DM | -13.99 | -38.06-10.08 | -0.195 | 0.245 |
| CAD | 32.80 | 3.75-61.84 | 0.380 | **0.028** |
| CKD | 1.18 | -12.98-15.34 | 0.029 | 0.866 |
| R=0.43, R^2^=0.19, Adjusted R^2^=0.11, F= 2.39, p= 0.087 | | | | |

DM: diabetes mellitus. CAD: history of coronary artery disease. CKD: chronic kidney disease (Cockroft-Gault eGFR <60 ml/min)

**Supplementary table 2:** Angiography-derived IMR values in LAD territory stratified according to time to coronary angiography

|  | **CAG ≤ 24h** | **CAG > 24h** | **p-value** |
| --- | --- | --- | --- |
| **NH-IMRangio LAD** | 52.6±19.9 | 57.8±19.7 | 0.485 |
| **AngioIMR** | 46.7±19.1 | 52±17.7 | 0.435 |
| **A-IMR** | 52.4±20.4 | 57.7±19.3 | 0.466 |

CAG: coronary angiography
